# HDGFI: Hierarchical Dual-Level Graph Feature Interaction Model for Personalized Recommendation

**DOI:** 10.3390/e24121799

**Published:** 2022-12-09

**Authors:** Xinxin Ma, Zhendong Cui

**Affiliations:** School of Computer and Control Engineering, Yantai University, Yantai 264005, China

**Keywords:** personalized recommendation, feature interaction, graph structure, dual-level graph feature interactions

## Abstract

Under the background of information overload, the recommendation system has attracted wide attention as one of the most important means for this problem. Feature interaction considers not only the impact of each feature but also the combination of two or more features, which has become an important research field in recommendation systems. There are two essential problems in current feature interaction research. One is that not all feature interactions can generate positive gains, and some may lead to an increase in noise. The other is that the process of feature interactions is implicit and uninterpretable. In this paper, a Hierarchical Dual-level Graph Feature Interaction (HDGFI) model is proposed to solve these problems in the recommendation system. The model regards features as nodes and edges as interactions between features in the graph structure. Interaction noise is filtered by beneficial interaction selection based on a hierarchical edge selection module. At the same time, the importance of interaction between nodes is modeled in two perspectives in order to learn the representation of feature nodes at a finer granularity. Experimental results show that the proposed HDGFI model has higher accuracy than the existing models.

## 1. Introduction

Personalized recommendation has been widely used in advertising [1], point of interest [2], e-commerce [3], and so on because it can solve the problem of online information overload. The core idea is to predict users’ preferences for items based on their historical purchase or click records [4]. Based on numerous user profiles, item attributes, and contextual information, feature interaction has become an important paradigm for recommendation systems. Learning the joint gain between features from complex feature interactions attracted a lot of attention both in industry and academia.

In order to separate feature interaction from complex manual settings, a series of methods have been proposed recently. Factorization machine (FM) [5] is a classic model that learns second-order feature interaction for the recommendation via the inner product of pairwise features. This model has triggered the proposal of a series of second-order feature interaction models. Attentional factorization machines (AFM) [6] and Field-aware factorization machines (FFM) [7] were constructed based on FM, which get a better performance compared with FM. These FM-based models can only capture second-order interactions and do not take into account the nonlinear higher-order interactions, which affect the performance of the models.

Deep neural networks (DNN) have the property of fitting any function and were widely used in the modeling of complex nonlinear problems with the development of deep learning. DNN-based models, such as deep factorization machine (DeepFM) [8], neural factorization machine (NFM) [9], and Wide&Deep [10], have been proposed to extend the low-order feature interactions to higher orders. However, although these models consider higher-order interactions between features, the interaction process between features is still implicit and uninterpretable. Moreover, they consider all feature interactions (including closely or negatively correlated), which can easily lead to important interactions being obscured by negatively correlated interactions. At the same time, the aggregation strategy models with deep learning represented by automatic feature interaction (AutoInt) [11], interpretable hierarchical attention interaction (InterHAT) [12], feature interaction graph neural network (Fi-GNN) [13], and graph factorization machine (GraphFM) [14] produce satisfactory results. They update the representation of field features and model higher-order interactions through aggregation strategies. However, aggregation strategy models also have two drawbacks. One is that they tend to select the last layer of feature representation or implicitly integrate the prediction results of different orders into the final prediction results without directly reflecting the influence of different orders on the final results. Second, although the attention mechanism can selectively convey or discard aggregated information, it still aggregates gain for some negative interactions, introducing noise to make training difficult.

To address these issues, we propose a hierarchical dual-level graph feature interaction recommendation model that adaptively selects the connections between nodes in different layers and explicitly generates the final recommendation results based on the graph representation of each layer. Firstly, the features of each sample are considered as nodes in the graph, and two connected nodes by an edge indicate that the two features interact. Secondly, because the interaction between features is not fixed, we adopt a hierarchical edge selection strategy for feature interaction selection, which can also be regarded as a graph-level dropout operation. Interactions with strong predictive gains are retained, and some unnecessary connections are filtered out. Thirdly, we design a local-level message passing and aggregation strategy for each layer’s graph structure and a global squeeze-excitation network (SENet) to capture representation fusion at different levels and iteratively obtain high-order feature interaction information. Finally, a graph-level representation is obtained to generate the final prediction score. The contributions of this paper are as follows.

- We consider several shortcomings of existing feature interaction models and use graph structure to model the interaction process between features, which increases the interpretability of feature interaction and improves the recommendation results of the model.
- We drop out the edges of the feature graph hierarchically, preserving the feature interactions that are most useful to the target node. The feature interaction process is modeled from local and global perspectives to obtain high interaction gain.
- We conducted experiments on three public datasets. The results show that our proposed model outperforms similar algorithms in terms of AUC and Logloss metrics.

## 2. Related Works

### 2.1. Feature Interaction Recommendation Model

Feature interaction, also known as feature combination, is a nonlinear transformation of the sample space by combining two or more features to achieve the goal of effective prediction for different feature combinations. Traditionally, feature interactions need to be generated manually, which requires specialized domain knowledge and is time-consuming and labor-intensive. FM [5] combines linear regression and feature decomposition models to learn both first and second-order interactions, which opens up a series of studies to automatically capture feature interactions. AFM [6] discriminates the importance of different feature interactions and introduces an attention module to extract the importance of different feature interactions on the final results. FFM [7] takes a feature domain perspective. Features of the same nature are grouped into one domain for modeling separately. Nevertheless, these FM-based models only consider second-order interactions and ignore higher-order interactions between features, limiting the predictive performance of the models.

With the development of deep learning, fusing DNN for feature interactions has gradually become a mainstream approach since DNN can fit arbitrary polynomials, which also contain polynomials of higher-order feature combination terms. Factorization-machine neural networks (FNN) [15] learn the higher-order feature interactions by connecting multiple fully connected layers using pre-trained FM as initialization parameters. DeepCrossing [16] adds multiple residual units to the multi-layer perceptron (MLP) layer to cross-combine the various dimensions of the features so that the model obtains more nonlinear and combinatorial feature information. Wide&Deep [10] is a combined linear regression and deep neural network model that can both learn high-frequency low-order features with a small number of parameters and make predictions for non-occurring samples. DeepFM [8] addresses the shortcoming that FM can only obtain second-order interactions but not higher-order interactions. DeepFM combines DNN and FM in parallel, where the higher-order feature interactions are performed in the DNN part, and the lower-order feature interactions are performed in the FM part. The bilinear feature interaction network (FiBiNET) [17] uses the SENet to dynamically learn the importance of features and uses a bilinear function to better model cross features. AutoInt [11] is inspired by Transformer [18] and uses a multihead self-attention mechanism to model the feature interaction process, which can capture higher-order feature crossover explicitly. InterHAT [12] adds residuals to AutoInt by adding residual connectivity and hierarchical attention to capture higher-order feature interactions.

### 2.2. Graph Neural Networks and Recommendation

The graph is a widespread data structure that can model a set of nodes and their relationships. Graph neural networks (GNN) is a deep learning representation model designed from graph structure, whose main idea is “aggregation” and “update.” “Aggregation” means collecting information from neighboring nodes as an aggregated representation of the neighborhood; “update” means using the neural network to update the representation of the nodes. Many variants of graph neural networks have been proposed recently, and we present some representative works. Graph convolutional networks (GCN) [19] consider the structural information of the graph and employs the convolutional aggregator to operate the first-order neighborhood. Graph sample and aggregate (GraphSAGE) [20] uses neighbor sampling for large-scale graphs and three new types of aggregators in the aggregation stage. Graph attention network (GAT) [21] concerns the differences between neighbors and uses attention-weighted aggregation to learn node representations. Gated graph neural networks (GGNN) [22] add a gated recurrent unit (GRU) to the update step to ensure convergence. The recommendation algorithm based on a graph neural network shows good application prospects due to the natural graph structure characteristics of the recommendation task.

Recently, GNN has been widely used in recommendation systems. Neural graph collaborative filtering (NGCF) [4] treats the collaborative relationship between users and items as a bipartite graph, which explicitly encodes higher-order collaborative signals. Knowledge graph attention network (KGAT) [23] combines the user-item graph with the knowledge graph, using a graph convolutional neural network to obtain the final node representation. Fi-GNN [13] used GNN in modeling feature interactions to aggregate information about neighboring nodes at first and then a GRU unit to update the node representation for more interpretability of the interaction process. 

However, the simple node aggregation method and fully connected graph structure method limit the capability of feature interactions. 

Our work attempts to model feature interactions from a finer granularity and select beneficial interactions to improve the expressiveness of the model.

### 2.3. Graph Structure Learning

The success of GNN is attributed to the use of graph structure and node attributes to learn downstream tasks. In many cases, the graph structure is incomplete and noisy. No natural graph structure can be used. From the perspective of representation learning, GNN computes node embedding by iteratively aggregating information from neighboring nodes. This mechanism may gradually process some noise in the original graph in the recursive process, worsening the representation quality of many nodes. In order to provide the best graph structure for downstream task learning, much graph structure learning has been carried out in recent years. The aim is to jointly learn and optimize the graph structures and corresponding graph representations. Graph structure modeling is an important core step in graph structure learning. It models edge connections, selects important edges to retain, and filters noisy edges. The current graph structure modeling works can be classified into three types: (1) Metric-based approaches. According to the network homogeneity assumption, edges tend to connect similar nodes [24]. Metric-based methods use a kernel function to compute the similarity between pairs of nodes. Adaptive graph convolutional neural networks (AGCN) [25] proposes a general and flexible graph convolutional network to learn a task-driven adaptive graph representation using distance metrics for different input graphs. Iterative deep graph learning (IDLG) [26] iteratively learns the graph structure and graph embedding to make the graph structure close enough to the downstream prediction task. (2) Neural approaches. Neural approaches use a deep neural network to infer edge weights. Graph learning-convolutional networks (GLCN) [27] obtain edge relationships between pairs of nodes through a single-layer neural network. Parameterized topological denoising network (PTDNet) [28] uses a multilayer perceptron to learn an adjacency matrix and remove task-independent edges to improve the robustness and generalization ability of GNN. (3) Direct approaches. In this way, the adjacency matrix is treated as a trainable parameter and optimized by backpropagation. Graph learning neural networks (GLNN) [29] adapts the graph topology to the input data by mixing efficient adjacency matrix properties sparsity and feature smoothness into the loss.

The graph feature interaction model represented by Fi-GNN models the relationship between feature fields as a fully connected graph without considering the interaction between feature node pairs. In this work, we use metric-based approaches to learn the graph structure and filter out the effective feature combination.

## 3. Proposed Model

### 3.1. Problem Definition

We formulate the recommendation task with necessary notations. $U=\{u_{1},u_{2},\ldots ,u_{M}\}$ and $V=\{v_{1},v_{2},\ldots ,v_{N}\}$ are the sets of M users and N items. $A=\{A^{1},A^{2},\ldots ,A^{J}\}$, $B=\{B^{1},B^{2},\ldots ,B^{K}\}$ and $C=\{C^{1},C^{2},\ldots ,C^{F}\}$ are the sets of J fields of user attributes, K fields of item attributes, and F fields of context, respectively. The user-item interactions are denoted as a matrix $Y_{M\times N}$, which $y_{uv}=1$ means user *u* has interacted with item *v* before, otherwise $y_{uv}=0$. Each user and item is associated with a list of attributes $A_{u}\in A$ and $B_{v}\in B$. In addition to user and item attributes, we denote a list of $C_{uv}\in C$ as context features. An instance can be represented as:

(1) $$x=[u,v,A_{u},B_{v},C_{uv}].$$

The purpose of the recommendation task is to design a prediction model which can be given an input sample x, the model can output a prediction probability $\hat{y}$ that the target user interacts with the candidate item.

### 3.2. Overview

**Figure 1:**
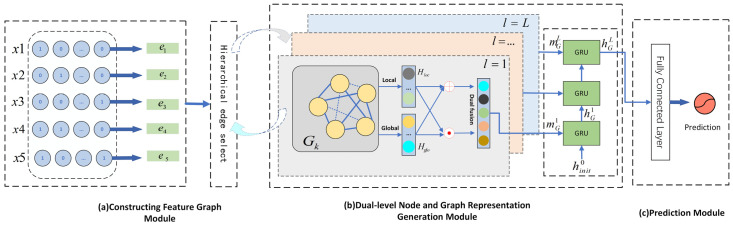
Overview of HDGFI.

The proposed Hierarchical Dual-level Graph Feature Interaction model is shown in Figure 1. Nodes are constructed for feature fields, and edges are constructed for interactions between features in the feature graph. The HDGFI model is composed of three main modules.

(1) Constructing Feature Graph Module. This module maps a high-dimensional and sparse raw feature to a low-dimensional dense vector representation. Each feature field is regarded as a node in the graph, and the edge connected with the node represents the interaction between features. A metric-based method is used to calculate the weight of edges and select important edges for connection.
(2) Dual-level Node and Graph Representation Generation Module. This module constructs two levels of feature interaction and fusion processes, which includes two components. One component is a local-level feature interaction that uses edge weights to update the node representation, and the other is a SENet component that captures important features at the global level.
(3) Prediction Module. This module uses the obtained feature interactions after each layer of node representation to calculate the final click probability.

### 3.3. Constructing Feature Graph Module

#### 3.3.1. Feature Graph Node Embedding

In the feature interaction recommendation task, the raw input features are multifield sparse features. For example, there are some feature fields in the instance of movie recommendation, such as {Language: English, Director: Christopher Nolan, Movie Genre: action movie, sci-fi movie…}. These multifield features cannot be directly input into DNN-based models. A conventional approach is to encode these fields into binary vectors, represented as:

(2) $$x=[x_{1},x_{2},\ldots ,x_{m}]=[\underset{field_{1}}{\underset{︸}{1,0,...0,}}\ldots ,\underset{field_{m}}{\underset{︸}{1,\ldots ,1,0}}],$$

where x is an input instance with an m field, $x_{m}$ is the m-th filed encoding representation, and *m* is the number of fields. Due to the large total number of features, the binary vector is high-dimensional and sparse, and the computational efficiency is not efficient. We convert the binary vector to a dense low-dimensional through a feature embedding layer to obtain feature embeddings.

(3) $$e_{i}=W_{i}^{emb}x_{i},$$

where $W_{i}^{emb}$ is an embedding matrix for field i that contains only one value and $x_{i}$ is a one-hot vector. For multi-valued feature fields such as movie genre, we extend Equation (3) and use the average of corresponding feature embedding vectors to represent the multi-valued feature field.

(4) $$e_{j}=\frac{1}{q}W_{j}^{emb}x_{j},$$

where $W_{j}^{emb}$ is an embedding matrix for multi-valued field *j*, *q* is the number of values, and $x_{j}$ is the multi-hot vector for the feature field. We can obtain the embedding vectors of these feature fields in an input instance:

(5) $$E=[e_{1},e_{2},\ldots ,e_{m}],$$

where $e_{m}\in {\mathbb{R}}^{d}$ is the embedding representation of the m-th feature field and d is the dimension of the embedding layer. We treat the embedded field embeddings as node representations in the feature graph, which also contains the first-order interaction information about the features.

#### 3.3.2. Hierarchical Edge Selection Layer

Previous work has constructed a fully connected graph by converting features into nodes and interactions between features into edges. However, not all interactions are useful for the final prediction, and some interactions can even play a weakening role. In order to capture the interaction pair with strong interaction gain and avoid the noise generated by unnecessary feature interaction, we learn the graph structure at each layer to predict the connection between feature nodes. However, the adjacency matrix is discrete, either connected or not connected. This makes it difficult to backpropagate the gradient. In order to solve this problem, inspired by an explicit sparse transformer [30], we generate a top-k beneficial adjacency weight matrix $A^{l}$ for each layer, where $A^{l}{}_{ij}$ represents the connection probability of node i and node j at layer l. Based on the rule of top-k selection, only the elements with the largest contribution are assigned probabilities in the architecture of hierarchical edge selection.

First, we express the similarity between node pairs as a score matrix *P*, which is mapped to a scalar through a two-layer fully connected network:

(6) $$P_{ij}^{l}=\sigma_{2}(W_{2}^{s}\sigma_{1}(W_{1}^{s}(e_{i}^{l}⊙e_{j}^{l})+b_{1})+b_{2}),$$

where $W_{*}^{s}$ and $b_{*}^{s}$ are trainable parameters; ⊙ is the element-wise product; $\sigma_{1}$ and $\sigma_{2}$ are LeakyReLU and Sigmoid activation functions, respectively.

**Figure 2:**
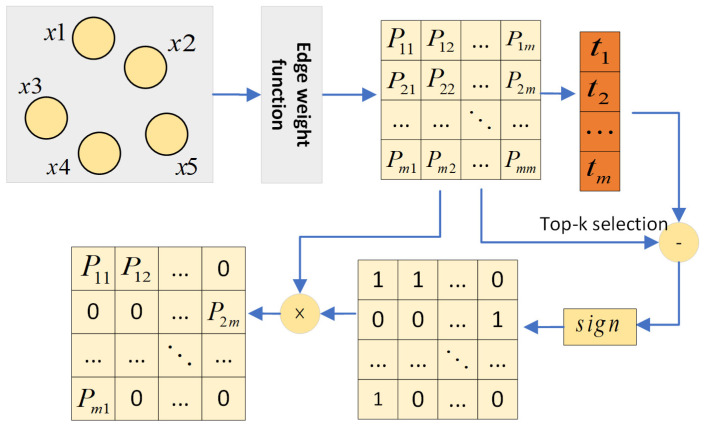
The architecture of hierarchical edge selection.

Then, we perform top-*k* selection on the attention score matrix via a masking operation $M\left(\cdot ,\cdot \right)$, as shown in Figure 2. In this way, the most important *k* feature interactions are preserved, while other irrelevant information is deleted. Calculate the beneficial adjacency matrix of the hierarchy using Equation (3):

(7) $$A_{ij}^{l}=M{\left(P^{l},k\right)}_{ij}=\left\{\begin{array}{l} P_{ij}^{l}\;P_{ij}^{l}\geq t_{i}\\ 0\;\;\;P_{ij}^{l}<t_{i} \end{array}\right.,$$

where $t_{i}$ is the t-th largest value of row i of attention score matrix *P*; the *k* is the hyper-parameter that controls the number of neighbors at each layer.

We keep the top-*k* values in the *P* matrix that are large and setting other values to 0 means that there is no interaction between the two features in the current layer.

### 3.4. Dual-Level Node and Graph Representation Generation Module

In this subsection, we use the feature graph constructed in the previous subsection to perform feature interaction between nodes.

#### 3.4.1. Local-Level Attention Messaging and Aggregation

**Figure 3:**
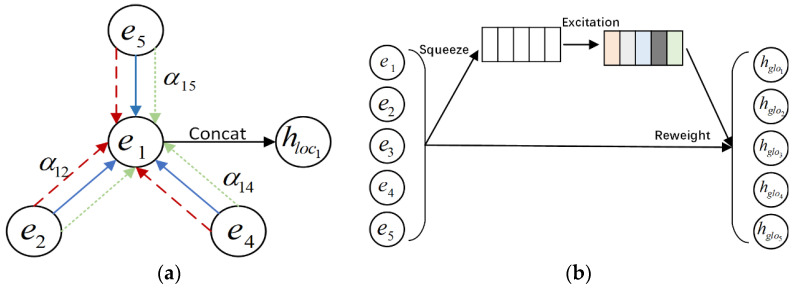
The architecture of dual-level node representation generation. (**a**) Local-level attention messaging and aggregation; (**b**) Global-level squeeze and excitation.

In the previous section, the interaction of two beneficial feature nodes is represented by an edge. We capture the higher-order interactions between features layer by layer through a graph attention network that captures the propagation and updates between information. The propagation process is shown in Figure 3a. Only the beneficial neighbor nodes of the target node of the current layer are propagated, which is different from the full-graph node propagation.

The attention coefficients can be computed as follows:

(8) $$c_{ij}^{\left(l\right)}=LeakyRelu\left(a^{T}\left(e_{i}^{l}⊙e_{j}^{l}\right)\right),$$

where $c_{ij}^{\left(l\right)}$ indicates the importance of node $v_{j}$ at l layer, LeakyRelu is the activation function and $a\in {\mathbb{R}}^{d}$ is the weight vector. After obtaining the importance of different neighboring nodes to the central node, we normalize the importance coefficients by the softmax function:

(9) $$\alpha_{ij}=\frac{\exp(c_{ij}^{l})}{\sum_{j\in Nei\left\{i\right\}}\exp(c_{ij}^{l})},$$

where $Nei\left(i\right)$ denotes the neighborhoods of node i, which is the set of beneficial interactions for node i; $\alpha_{ij}$ is the attention weight when aggregating neighbor node j.

After obtaining the attention weights of the central node to the neighboring nodes, we use a multi-head attention fusion mechanism to update the representation of the target node, which is calculated as follows:

(10) $$h_{{}_{loc_{i}}}^{l}=\overset{U}{\underset{u=1}{\left|\right|}}\sigma \left(\sum_{j\in N_{i}}A_{ij}^{l}\alpha_{ij}^{u}W^{u}\left(e_{i}^{l}⊙e_{j}^{l}\right)\right),$$

where || is the concatenation; u is the number of the attention heads; $\alpha_{ij}^{u}$ is the attention score of the *u*-th head; $W_{}^{*}$ is the linear transformation matrix. After the above operations, we can obtain the node’s local-level representation $H_{loc}^{l}=\left[h_{loc_{1}}^{l},h_{loc_{2}}^{l},\ldots ,h_{loc_{m}}^{l}\right]$.

#### 3.4.2. Global-Level Squeeze and Excitation

The local-level node fusion module focuses on the interaction between node pairs but does not pay attention to the importance of nodes in the entire feature field. Different feature fields have different importance to the target task. In film recommendation, gender, age, and genre are usually more important than user occupation and region. We hope to dynamically increase the weight of important features while reducing the weight of unnecessary features. SENet has achieved great success in image classification tasks, and it can model the interdependence between convolutional feature channels to improve the representation ability of the network. In order to retain the previously learned combined features and dynamically capture the importance of feature fields, we design a squeeze and excitation module at the global level for residual connection, as shown in Figure 3b.

**Squeeze.** The step requires summary statistics of the embeddings fused at the local level for each field. We use average pooling to convert the representation of a node to a scalar.

(11) $$s_{i}=\frac{1}{d^{\prime }}\sum_{t=1}^{d^{\prime }}e_{i}^{l},$$

where $s_{i}$ represents the global information about the *i*-th feature representation. We compress the representation of each field into a statistical vector $S=\left[s_{1},s_{2},\ldots ,s_{m}\right]$.

**Excitation.** This step uses two fully connected layers, one for dimensionality reduction and one for dimensionality increase, through a reduction ratio hyper-parameter. The dynamic global weight of each feature field can be calculated as follows:

(12) $$att_{glo}=\sigma_{3}\left(W_{2}^{g}\sigma_{3}\left(W_{1}^{g}S\right)\right),$$

where $att_{glo}$ is the global-level attention score; $W_{1}^{g}\in {\mathbb{R}}^{m\times \frac{m}{r}}$ and $W_{2}^{g}\in {\mathbb{R}}^{\frac{m}{r}\times m}$ are the learning parameters; $\sigma_{3}$ is the Tanh activation functions.

**Reweight.** The last step is a reweight step which is called re-scale in paper [31]. We recalculate the node vector of global influence by the global attention score as follows:

(13) $$H_{glo}^{l}=att_{glo}⊙E^{l}=[att_{glo_{1}}\cdot e_{1}^{l},att_{glo_{2}}\cdot e_{2}^{l},\ldots ,att_{glo_{m}}\cdot e_{m}^{l}].$$

#### 3.4.3. Dual-Level Node Embedding Fusion

Once obtained the node representations at the local level and global levels, we used a bilinear-cross aggregation function to improve the representation of node interactions as follows:

(14) $$e_{i}^{l}=W_{\varphi }^{l}[h_{loc_{i}}⊕h_{glo_{i}},h_{loc_{i}}⊙h_{glo_{i}}],$$

where ⊕ and ⊙ are the elementwise addition and the Hadamard product, and $W_{\varphi }^{l}$ is the trainable matrix. The elementwise addition operation can convey more information from similar features, and the sum operation can highlight features with larger accumulated values.

#### 3.4.4. Graph Representation Readout

After the above operations, the node representation has been updated. In other words, each feature field node is neighborhood aware. We design a readout operation of graph representation to dynamically capture the graph embedding of each layer after the interaction. The readout operation is shared by all time steps. For all nodes in the graph, the average pooling is used to represent global graph message vectors. The formula is as follows.

(15) $$m_{G}^{l}=\frac{1}{m}\sum_{i=1}^{m}e_{i}^{l}.$$

Then, the current layer global message vectors $m_{G}^{l}$ and graph vectors of the previous layer readout operation $h_{G}^{l-1}$ are sent to the GRU to update the graph feature $h_{G}^{l}$:

(16) $$h_{G}^{l}=GRU(m_{G}^{l},h_{G}^{l-1}).$$

Initialized graph features $h_{G}^{0}$ is the sum of the initial embedding addition. Through the readout operation, the graph features are updated at each time step.

### 3.5. Prediction and Optimization

Following the feature graph dual-level node and graph representation generation module, we can obtain the graph vector of the last layer. We employ a fully connected layer parameterized by *θ* and the sigmoid activation function to obtain final predictions as follows:

(17) $$\hat{y}=sigmoid\left(\theta^{T}\cdot h_{G}^{L}\right).$$

To train our model, we define the loss function as the cross-entropy of the prediction and the ground truth.

(18) $$L=-\frac{1}{N}\sum_{i=1}^{N}\left(y_{i}\log\left(\hat{y_{i}}\right)+\left(1-y_{i}\right)\log\left(1-\hat{y_{i}}\right)\right),$$

where $y_{i}$ is the ground-truth value of the i-th training instance, $\hat{y_{i}}$ is the prediction of our model, and N is the number of training instances. At the same time, we use backpropagation to continuously update the trainable parameters.

## 4. Experiments

### 4.1. Experiment Setup

#### 4.1.1. Datasets

To evaluate the performance of our proposed model, we chose three publicly available datasets. The statistics of the datasets are summarized in Table 1.

**The dataset KKBox** is a professional digital music information service software. Each user can find their favorite music in the shortest time. The features of the data set include 13 feature domain information such as song ID, songwriter name, user gender, and user age.

**The dataset Frappe** is a context-aware app discovery tool. Each record contains the user ID and APP ID, as well as eight context information, including weather, city, etc. A target value of 1 indicates that the user has used an APP under context information.

**The dataset MovieLens-1M** is a dataset of the user ratings of movies. We expanded the dataset by collecting information on the directors, actors, tags, etc., of the movies from the IMDB website. We consider samples with more than three ratings as positive ratings.

#### 4.1.2. Evaluation Metrics

We use the following two metrics for model evaluation: AUC (Area Under the ROC curve) and Logloss (cross-entropy).

**AUC** is the area bounded by the coordinate axis under the ROC (Receiver Operating Characteristic Curve). It ranges from 0 to 1 and is a performance indicator to measure the quality of a classifier. A classifier with a larger AUC has better results.

**Logloss** is the most important classification metric based on probability. For any given problem, a lower value of Logloss implies a better prediction.

#### 4.1.3. Baselines

We compare the proposed model with four types of baselines: (1) A. Linear models for modeling first-order Raw Feature Interactions. (2) B. FM and FM-based second-order feature interaction model. (3) C. Deep learning-based models capable of capturing higher-order interactions. (4) D. Graph-based models for characterizing features and feature interactions with nodes and edges.

**LR** (A) [32] refers to linear logistics regression, which can only model linear interactions.

**FM** (B) [5] is one of the classical models for an attribute-aware recommendation based on modeling the interaction of each feature with an inner vector product.

**AFM** (B) [6] is a variant of FM that uses attentional mechanisms to distinguish the importance of feature interactions.

**FFM** (B) [7] adds the concept of the field on the basis of FM, taking into account the similarities and differences between different features.

**NFM** (C) [9] captures second-order feature interactions using feature interaction pooling instead of splicing operations and captures higher-order interactions with DNNs.

**DeepFM** (C) [8] uses FM to feature low-order combinations while using MLP part to feature high-order combinations. 

**FiBiNet** (C) [17] combines the importance of features and designs a new way of bilinear feature interaction between feature fields.

**Fi-GNN** (D) [15] constructs each sample into a feature graph, with each node as a feature field, and models the feature interactions using a graph neural network.

**GraphFM** (D) [14] uses a graph neural network to solve the defects of FM and treats feature interaction from the perspective of graph structure learning.

#### 4.1.4. Hyper-Parameter Settings

All the experiments are performed on the Pytorch platform and each is randomly split each dataset into training, validation, and test sets with a ratio of 6:2:2. All model feature field dimension vectors are set to 16, and the number of hidden units is 32. The learning rate is set to 0.001 for KKBox and MovieLens-1M and 0.005 for Frappe datasets. The L2 regularization factor is 0.0001. For Autoint, Fi-GNN, and GraphFM models, we set three layers. We use binary cross-entropy loss as a loss function and use Adam to optimize these models.

To ensure a fair comparison, we run all experiments by five folds cross-validation and report the averaged results. We perform statistical significance test results (through Wilcoxon signed rank test) to verify the statistical significance in comparing our method with the best baseline methods.

### 4.2. Overall Performance

Based on the experimental setup described above, we compared HDGFI with other baseline models. As illustrated in Table 2, we observed the following results:

(1) LR showed the worst performance as a linear model to capture the first-order interactions of features. Higher-order interactions and nonlinear relationships between features are very important for prediction tasks.

(2) The effect of the second-order feature interaction model based on FM is better than that of LR, which indicates that pairwise feature interactions can improve prediction accuracy. AFM outperforms the baseline model FM, showing that attention can avoid the noise caused by the addition of useless feature intersections. 

(3) Higher-order interaction models based on deep learning perform better than lower-order interaction models, demonstrating that DNNs can capture higher-order implicit feature interactions.

(4) Graph-based feature interaction models generally outperform low-order and deep interaction models. The reason is that the graph structure enables feature interaction in an explicit way, transforming the interaction into message propagation and representation update between feature graph nodes. At the same time, the graph structure is also explanatory of the interaction between features.

(5) Our proposed HDGFI model shows the best performance on both datasets, with the *p*-value of all metrics rejecting the null hypothesis with a level of significance α=5%. More precisely, HDGFI has the highest AUC values (0.82278, 0.97894, and 0.91113) and the lowest Logloss values (0.51555, 0.16495, and 0.37871) on KKBox, Frappe, and MovieLens-1M. Previous works [8,11] have demonstrated that improvements in AUC metrics at the 0.001 level are important for click-through rate prediction tasks. The possible reason why HDGFI is superior to the baseline model is that we have conducted hierarchical beneficial interaction selection and filtered out unnecessary noise interactions. Meanwhile, the node interaction at the local pair-wise level and the feature importance selection at the global graph level are considered.

**Table 2:** Overall accuracy comparison in the three datasets and significance test. The bold value marks the best one in each column.

Dataset Model	KKBox		Frappe		MovieLens-1M	
	AUC	Logloss	AUC	Logloss	AUC	Logloss
LR(A)	0.76647	0.57593	0.93565	0.28721	0.86949	0.43775
FM(B)	0.78961	0.55487	0.96571	0.20912	0.89104	0.42229
AFM(B)	0.79868	0.54858	0.96534	0.21947	0.88224	0.42861
FFM(B)	0.79758	0.54323	0.96871	0.19901	0.89563	0.40881
NFM(C)	0.80979	0.53088	0.97283	0.20717	0.89975	0.40351
DeepFM(C)	0.81439	0.52556	0.97551	0.18532	0.90617	0.38856
FiBiNet(C)	0.81783	0.52207	0.97554	0.18061	0.90628	0.39021
Fi-GNN(D)	0.81831	0.52033	0.97541	0.18431	0.90668	0.38755
GraphFM(D)	0.82013	0.51872	0.9764	0.17824	0.90782	0.38378
HDGFI(ours)	**0.82278**	**0.51555**	**0.97894**	**0.16495**	**0.91113**	**0.37871**
*p*-value	2.82%	2.82%	0.9%	0.9%	0.9%	1.62%

### 4.3. Hyper-Parameter Study

We conduct two hyper-parameter experiments to evaluate the model, including the number of neighbor samples and the global-level reduction ratio. The hyper-parameter results based on the division proportion 6:2:2 of the raw dataset are shown in this section.

#### 4.3.1. Influence of Neighborhood Sampled Size

**Figure 4:**
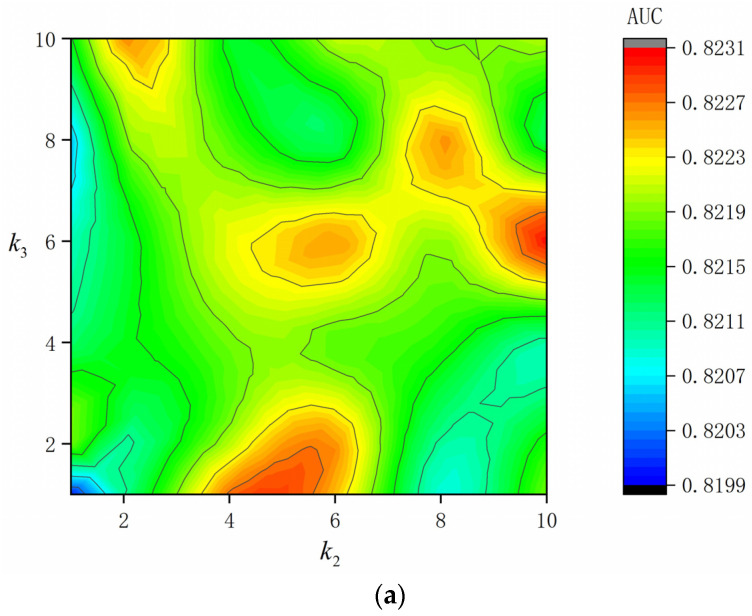

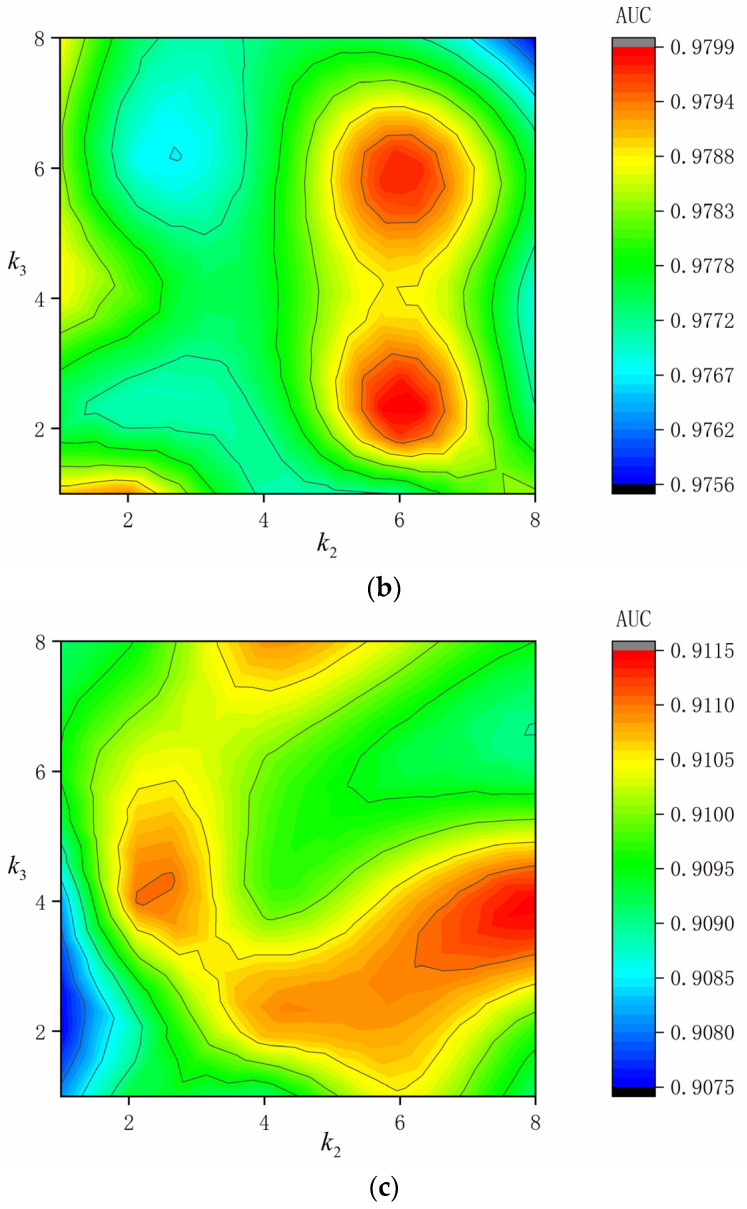
Influence of neighborhood sampled size. (**a**) Influence of neighborhood sampled size on KKBox; (**b**) Influence of neighborhood sampled size on Frappe. (**c**) Influence of neighborhood sampled size on MovieLens-1M.

The number of neighbor samples at each layer is an important hyper-parameter for our model. We fixed the number of samples in the first layer as the number of feature fields, which means that we established a fully connected graph. Because in the first layer, we used to capture the interaction relationship between each node pair as much as possible. When a different number of samples in the second and third layers is selected, the results are shown in Figure 4. $k_{2}$ and $k_{3}$ are the number of neighbor samples of the target node in the second and third layers, respectively. On the KKBox dataset, we can observe that the performance is optimal when the $k_{2}$ = 10, $k_{3}$ = 6, and suboptimal when $k_{2}$ = 6, $k_{3}$ = 2. The performance is the worst when one neighbor node is selected at each layer. On the Frappe dataset, the model performance peaks with $k_{2}$ = 6, $k_{3}$ = 2, or 6. On the MovieLens-1M dataset, the best effect is achieved when $k_{2}$ = 8 and $k_{3}$ = 4.

#### 4.3.2. Influence of Reduction Ratio

**Figure 5:**
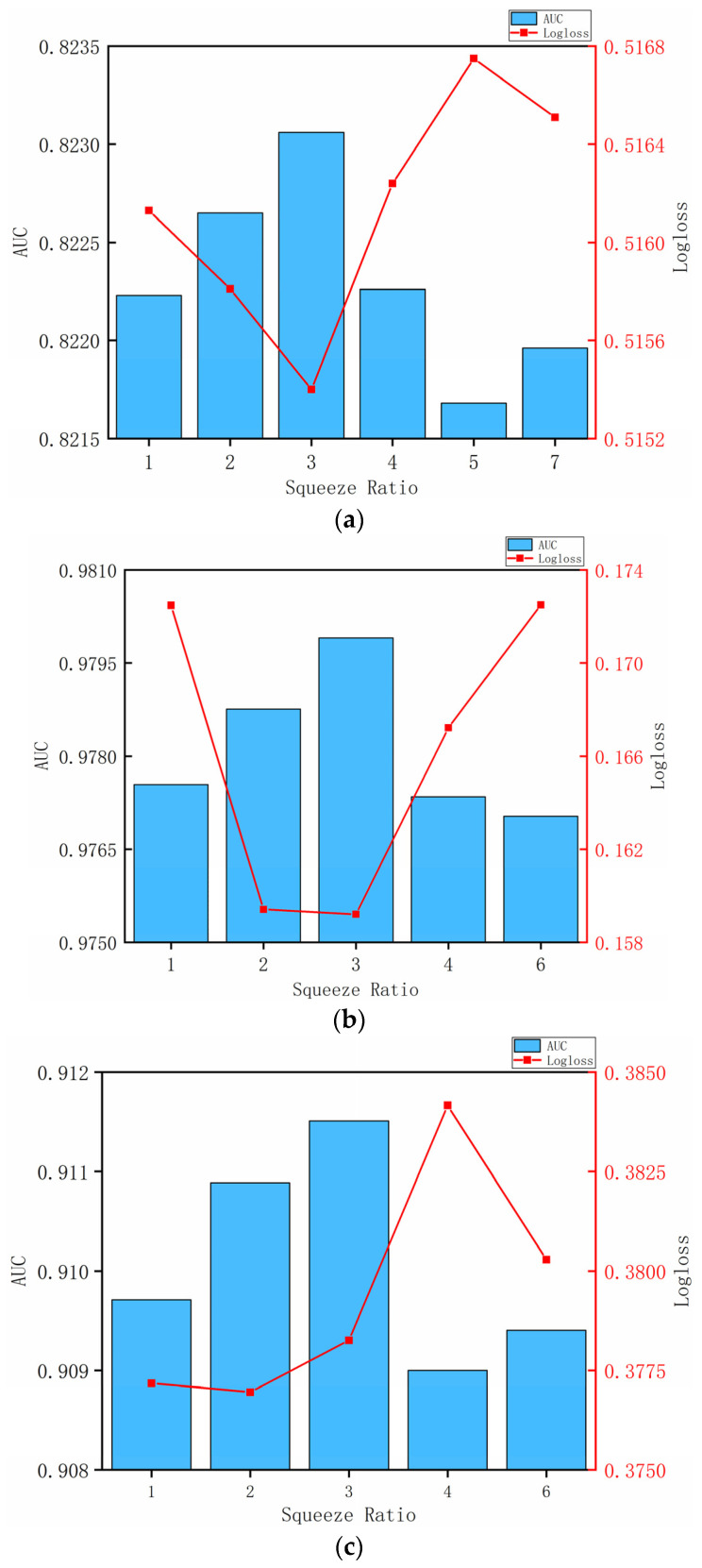
Influence of different reduction ratios. (**a**)Influence of different reduction ratios on KKBox; (**b**) Influence of different reduction ratios on Frappe. (**c**) Influence of different reduction ratios on MovieLens-1M.

As shown in Figure 5a–c, the best reduction ratio for KKBox and Frappe is 3. MovieLens dataset achieves the optimal results of logloss and AUC on 2 and 3, respectively. Considering that there is little difference in the number of feature fields between the three datasets, we can infer that squeeze from the original number of feature fields to 30–40% and excitation is the best effect.

### 4.4. Ablation Study

In this section, we investigate the impact of different components in the model on performance. Our proposed model has two major improvements: (1) We use local-level attention messaging to capture feature interactions between pairs of beneficial feature nodes. (2) We use the global-level squeeze and excitation module to retain the feature combination information learned in the previous layer. To evaluate the effectiveness of these two levels, we created two variant models to perform ablation experiments.

HDGFI_L: HDGFI without local-level feature interaction, i.e., uses DNN to capture high-order interactions. 

HDGFI_G: HDGFI without global-level dynamic importance selection.

As shown in Table 3, the performance of the two variant models degraded comparing with the HDGFI model, which indicates that both the global level and local level are necessary.

In order to explore the influence of more details on the model, we conducted ablation experiments about edge selection strategy and bilinear fusion.

HDGFI_E: HDGFI, without hierarchical edge selection, construct a completely connected feature interaction graph.

HDGFI_B: HDGFI without bilinear-cross aggregation.

The following conclusions can be drawn from Table 4. 

(1) HDGFI_L compared with HDGFI. Pair-level interactions between nodes can reflect the importance of local interaction. Compared with the DNN implicit capture high-order interaction method, modeling features as nodes and iteratively updating the node representation can better explicitly model the interaction between features.

(2) HDGFI_G compared with HDGFI. The global-level feature interaction can adaptively capture the importance of node features learned in previous layers in a residual way so that each layer can learn more accurate node representation. This can help to better perform the interactive feature selection of the next layer and learn the feature graph representation of the current layer.

(3) HDGFI_E compared with HDGFI. The feature field nodes are modeled as fully connected graphs, and the edge weights are fixed from the beginning. This approach preserves all feature interactions to a certain extent, but unnecessary interactions have a negative impact on the final prediction results. The edge selection operation can only focus on strong feature interactions, thus filtering out relatively unimportant interactions.

(4) HDGFI_B compared with HDGFI. The bilinear-cross aggregation function captures the fine-grained interaction between the two levels of node representation better than simple vector concatenation.

**Table 4:** Performance comparison of HDGFI with HDGFI_E and HDGFI_B.

Dataset Variants	KKBox		Frappe		MovieLens-1M	
	AUC	Logloss	AUC	Logloss	AUC	Logloss
HDGFI_E	0.82149	0.51716	0.97741	0.16437	0.90939	0.38051
HDGFI_B	0.82111	0.51738	0.97738	0.17088	0.90975	0.38025
HDGFI	**0.82278**	**0.51555**	**0.97894**	**0.16495**	**0.91113**	**0.37871**

## 5. Conclusions

Due to the increasing maturity of deep learning technology, automatic feature engineering instead of manual feature engineering received wide attention. Automatic capture of fine-grained feature interactions has become one of the focuses of recommendation systems, which attracted extensive attention from academia and industry. In this paper, we propose a hierarchical dual-level graph feature interaction recommendation model. The proposed model uses a hierarchical edge-selection module to filter out unnecessary interactions and reduce the interference of noise interactions on prediction. At the same time, the modules of local message passing, and global important feature selection are proposed to capture the interaction relationship between features from two perspectives. Experimental results proved that the HDGFI model is effective and outperforms other state-of-the-art baselines in terms of accuracy on three public datasets. In the future, we will explore adaptive learning of the most beneficial number of feature interactions for each layer and more advanced ways to model feature interaction graphs.

## Figures and Tables

**Table 1:** Statistics of the datasets.

Dataset	Num of Fields	Num of Features	Instances
KKBox	13	92,247	7,377,418
Frappe	10	5382	288,609
MovieLens-1M	10	22,100	1,149,238

**Table 3:** Performance comparison of HDGFI with HDGFI_L and HDGFI_G.

Dataset Variants	KKBox		Frappe		MovieLens-1M	
	AUC	Logloss	AUC	Logloss	AUC	Logloss
HDGFI_L	0.80779	0.53411	0.97331	0.19246	0.90016	0.39976
HDGFI_G	0.81679	0.52293	0.97598	0.17051	0.90914	0.38133
HDGFI	**0.82278**	**0.51555**	**0.97894**	**0.16495**	**0.91113**	**0.37871**

## Data Availability

KKBox data is available from https://www.kaggle.com/competitions/kkbox-music-recommendation-challenge. Frappe data is available from http://baltrunas.info/research-menu/frappe. MovieLens-1M data is available from http://grouplens.org/datasets/movielens/.
